# A nanosol SERS/RRS aptamer assay of trace cobalt(ii) by covalent organic framework BtPD-loaded nanogold catalytic amplification†

**DOI:** 10.1039/d1na00208b

**Published:** 2021-05-05

**Authors:** Guiqing Wen, Yang Xiao, Shuxin Chen, Xinghui Zhang, Zhiliang Jiang

**Affiliations:** Key Laboratory of Ecology of Rare and Endangered Species and Environmental Protection (Guangxi Normal University), Ministry of Education Guangxi China zljiang@mailbox.gxnu.edu.cn; Key Laboratory of Environmental Pollution Control Theory and Technology Guilin 541004 China

## Abstract

The determination of heavy metal ions has always been a hot topic in the field of environmental analysis. In this study, a new covalent organic framework-loaded gold nanoparticle (AuCOF) nanocatalytic amplification signal strategy was developed to determine trace Co^2+^ in water. The COF of BtPD was synthesized from 1,3,5-benzene tricarboxaldehyde and *p*-phenylenediamine, and a new kind of AuBtPD nanosol was prepared by reduction of HAuCl_4_ to AuNPs on the BtPD carrier. It has strong catalysis of the new indicator reaction of sodium formate reducing HAuCl_4_ to AuNP sol with strong resonance Rayleigh scattering (RRS) at 370 nm and surface enhanced resonance Raman scattering (SERS) activity at 1614 cm^−1^ in the presence of a Victoria blue 4R (VB4R) molecular probe. Combining the nanocatalytic reaction to amplify the dual-scattering signals and specific aptamer (Apt) of cobalt ions, a new, fast, stable, sensitive and specific dual mode method for detecting Co^2+^ was established; the RRS signal *I*_370nm_ and SERS signal *I*_1614cm^−1^_ show a linear relationship with the concentration of 0.033–1 nmol L^−1^ Co^2+^ and with a limit of detection (LOD) of 0.02 nmol L^−1^. The two methods have been applied to the determination of Co^2+^ in industrial wastewater, tap water and river water, and the results are satisfactory.

## Abstract 2

A sensitive gold nanosol SERS/RRS aptamer assay of cobalt(ii) based on AuBtPD catalytic amplification.

## Graphical abstract

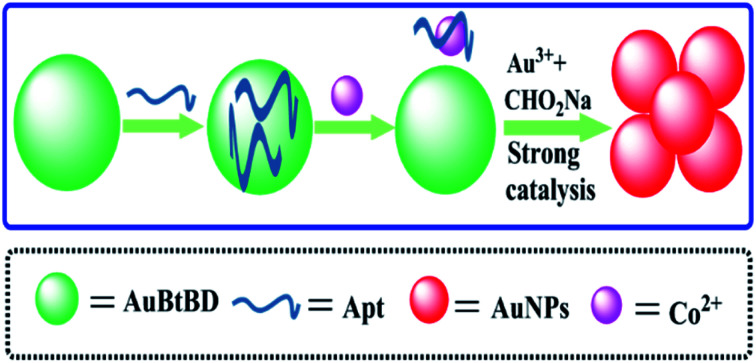

## 1. Introduction

The covalent organic frameworks (COFs) are a group of porous organic materials linked by covalent bonds. Most of these porous organic materials are synthesized by two or more units containing rigid aromatic hydrocarbons. Because of their high porosity, orderly arrangement, low density and modifiability, they have a broad application prospect in gas storage, adsorption, catalysis, chemical sensing, optoelectronics and other fields, and their synthesis and application are favored by people.^1,2^ Wang *et al.* prepared COF-LZU1 by loading Pd ions in a COF, which had excellent catalytic efficiency of up to 95% for the Suzuki coupling reaction and showed good reusability.^3^ Zheng *et al.* prepared a TPPA-1 COF solid phase microextraction (SPME) fiber using a high efficiency *in situ* synthesis strategy and successfully realized the detection of progesterone, testosterone and dehydroepiandrosterone in human serum by HPLC-MS/MS, with a LOD of 0.75 ng mL^−1^ and a linear range of 0.100–100 ng mL^−1^.^4^ Huang *et al.* modified the COF into carbon fiber paper to make it functional^5^ and realized the efficient trapping for ˙OH in air. The detection range was from 3.0 × 10^7^ to 6.0 × 10^11^ mol cm^−3^ and the LOD was 4.1 × 10^6^ mol cm^−3^. Zhang *et al.* synthesized COF materials from 1,3,5-trimethylphloroglucinol (TP) and benzidine (BD)^6^ and successfully prepared PTNP-COF composites with improved electroactivity by *in situ* growth of Pt nanoparticles on the surface of the COF for the electrochemical detection of 0.018 μmol L^−1^ tanshinol. Pan *et al.* doped silver nanoparticles in a COF as a catalyst, which enhanced the catalysis of copper tartrate-glucose to produce Cu_2_O particles and was coupled with the aptamer reaction to detect melamine by RRS.^7^ However, reports on the porous COF-loaded AuNPs as nanocatalysts and their application in SERS and RRS dual-mode analysis are still relatively rare.

Rayleigh scattering is a kind of elastic scattering and Raman scattering is a kind of inelastic scattering. Resonance Rayleigh scattering (RRS) has the advantages of high sensitivity, convenience, and speediness and is widely used in many kinds of analysis.^8^ Wen *et al.* added human chorionic gonadotropin (HCG) to the aggregated silver nano-sol that reacted with polypeptides and made the silver nanoparticles disperse since the polypeptides bind to HCG preferentially. The dispersed nanosilver can catalyze the H_2_O_2_–HAuCl_4_ reaction and produce AuNPs that have strong RRS/SERS signals. The new method can detect 0.05–20 ng mL^−1^ hormone.^9^ Han *et al.* prepared a MnO_2_ nanosheet with strong RRS and absorption signals.^10^ An optical dual-mode method for the detection of glutenin was established with detection ranges of 0.1–20 μmol L^−1^ and 2–200 μmol L^−1^, respectively, and a LOD of 0.033 μmol L^−1^ and 0.67 μmol L^−1^, respectively. Surface enhanced Raman scattering (SERS) usually occurs on the rough surface of gold, silver and copper. When some molecules are adsorbed on the surface of this kind of metal, the Raman scattering intensity will be greatly enhanced, up to 10^4^ to 10^6^ times, attracting great interest of researchers in various fields.^11^ As a high sensitivity and fast detection method, SERS has great potential in all kinds of trace detection, and is widely used in environment, biology, food inspection and other aspects.^12,13^ Lei *et al.* created a graphene wrapped Ag array based flexible membrane sensor to detect malachite green, which is a banned drug in aquatic products,^14^ with a LOD of 2.7 × 10^−11^ mol L^−1^. Xiang *et al.* prepared nano-sized silver with sodium alginate as a reducing agent.^15^ Carbimazole with nano-sized silver as a substrate had a strong SERS signal. When Cr(vi) was added, the SERS signal decreased due to the redox reaction between Cr(vi) and carbimazole, so a method for the determination of 1.71–171 × 10^−9^ mol L^−1^ Cr(vi) was established. Ai *et al.* synthesized flower-shaped silver nanoparticles by reducing silver nitrate with ascorbic acid.^13^ The silver nanoparticles had high SERS activity and were used as the substrate to detect 10^−9^ mol L^−1^ rhodamine 6G. The dual-mode analysis method is a kind of an analysis sensor model designed to obtain multiple states or properties of the same measured object bidirectionally in the same analysis model. Compared with the single feedback analysis method, this method combines the advantages of the single analysis model, solves the problem of crosstalk when using several different types, and has higher selectivity and accuracy. In recent years, with the deepening of research, the dual-mode analysis method has been developed and applied in biology, environment and other aspects.^16^ Lu *et al.* developed a dual-mode pH-responsive enzyme-linked immunosorbent assay using curcumin as the reporter molecule, based on the fluorescence effect and chromochromic effect induced by fluorescence resonance energy transfer and the color change effect, to detect cardiac troponin.^17^ Liang *et al.* found that BiH_3_ gas can be absorbed by graphene oxide and lead to the reduction of graphene oxide (GO), and the surface plasmon RRS energy of GO transfers to I^3−^ easily, which leads to the quenching of RRS.^18^ I^3−^ and Victoria blue B could form associated molecules to change SERS signals, so a dual mode method for the RRS/SERS detection of Bi was established.

An aptamer (Apt) is a short DNA or RNA oligonucleotide that is selected from the nucleic acid library by systematic evolution of ligands by exponential enrichment, and it binds to specific ligands with high efficiency and specificity. It not only has the characteristics of general antibodies, but also has the advantages of wide application, easy synthesis, easy modification and so on. It has been widely used in biomedical, environmental and food detection and other aspects.^19–21^ Romero-Reyes *et al.* developed an Apt-functionalized membrane that can effectively remove the small molecule pollutant bisphenol A from water and can be regenerated for a variety of uses.^22^ This research provides a new option for removing and recycling certain small molecules from water. Yi *et al.* found that the ofloxacin aptamer could be recognized and inserted by the fluorescent inserter SYBR green I (SG-I) and generate a strong fluorescence signal.^23^ With the addition of ofloxacin, the Apt was combined with ofloxacin and SG-I was released into the solution again, resulting in the weakening of the fluorescent signal of the system, which could selectively detect 1.1–200 nmol L^−1^ ofloxacin. Wu *et al.* used a label-free signal-on fluorescence aptasensor based on the Tb^3+^–Apt as a probe to detect the T-cell acute lymphoblastic leukemia cell line (CCRF-CEM).^24^ CCRF-CEM could combine with the Tb^3+^–Apt to form a Tb^3+^–Apt–CEM complex and cause fluorescence changes. The fluorescence signal was linear with the concentration of cells, with a linear range of 5–5 × 10^6^ cells per mL, and the LOD was 5 cells per mL. This method is rapid, economic, and has a good specificity. Zhou *et al.* used a kind of Apt to coat the surface of AuNPs to determine 1–500 nmol L^−1^ silver ions colorimetrically.^25^

Cobalt is widely used in industry, as an important raw material for pigments, heat-resistant alloys, hard alloys and other materials, and is closely related to human life. It is an indispensable trace element in the human body and an important component of vitamin B12, which is inseparable from the production of erythrocytes. Lack of cobalt can easily cause anemia and other related symptoms, but excessive intake of cobalt can lead to cardiovascular, nervous system and skin related diseases. Long-term exposure to cobalt-containing dust is an important cause of chronic pulmonary fibrosis, and cobalt affects biological DNA repair, causing various potential adverse effects on human health.^26^ Therefore, the detection of cobalt in the environment is particularly important for the further study of the biological mechanism, pathogenic mechanism and environmental effects of trace cobalt. D. Vashisht *et al.* reported a coumarin based azomethine colorimetric probe.^27^ This probe can selectively combine with Co^2+^ to form a complex, due to intramolecular charge transfer (ICT), and the color of the solution changes from light yellow to yellowish red. A method for the determination of Co^2+^ in water was established according to the change of the ultraviolet signal. The detection range of this method is 0–90 μmol L^−1^ and the LOD is 7.09 μmol L^−1^. In recent years, new nanomaterials have been applied to the detection of cobalt ions. Wang *et al.* prepared sulfur quantum dots with good photoluminescence intensity through a “top-down” approach.^28^ The sulfur quantum dots would aggregate and quench fluorescence when Co^2+^ was added to the solution. A method for detecting Co^2+^ was established through the change of the fluorescence signal, and the method had good linearity when the concentration of Co^2+^ was 0–90 μmol L^−1^. Wissutaboonta *et al.* prepared a Co^2+^ fluorescence sensor based on nitrogen–sulfur co-doped graphene quantum dots detection with a LOD of 1.25 μmol L^−1^.^29^ Zhao *et al.* synthesized polymer dots (PDs) with stable fluorescence effect *via* a microwave-assisted method.^30^ The PDs have high ion concentration resistance and extreme pH resistance, and the addition of Co^2+^ in the solution can quench the fluorescence of PDs. The concentration of Co^2+^ is linear with a fluorescence signal at 3.4–50 and 46.7–600 μmol L^−1^. The LOD of this method is 1 μmol L^−1^. The solutions of PD drops on quartz glass can also produce obvious signal changes, providing a reference method for on-site detection. However, there are still a few reports on the highly sensitive detection of Co^2+^ by combining RRS/SERS spectra with an Apt. Dionysia Tsoutsi *et al.* prepared a kind of silver nanoparticles functionalized with dithiocarbamate salt.^31^ This material had a high affinity for Co^2+^, and Co^2+^ made the SERS spectrum of the material change, which could realize the detection of a minimum of 60 ppt of Co^2+^ with a linear range of 0–5.9 ppb. Liu *et al.* found that the complex produced by the reaction of Co^2+^ with PAN would further form into an ion-association complex with sodium dodecyl benzene sulfonate under buffering conditions. The RRS intensity of the solution was enhanced, and a cobalt detection RRS method was established; the detection limit was 2.68 × 10^−9^ g mL^−1^ and the line width was 0–5.0 × 10^−7^ g mL^−1^.^32^ Qiu *et al.* found that Co^2+^ with thiocyanate and neutral red (NR) could generate a characteristic RRS spectral signal in the presence of PVA-124, which was proportional to the concentration of Co^2+^ within 0–0.48 μg mL^−1^. The minimum detection limit was 4.4 × 10^−5^ μg mL^−1^.^33^ However, such RRS or SERS methods for detecting cobalt ions have low sensitivity and are susceptible to interference. The COF is a good catalyst and can also be used as an ideal carrier. In this paper, a novel preparation procedure of highly stable AuBtPD nanosol was explored based on porous BtPD as the carrier and trisodium citrate as the reducer. Moreover, a new highly sensitive dual-mode scattering method was developed for the determination of Co^2+^ coupled with the strongly catalytic amplification reaction and specific Apt reaction. Compared with other RRS/SERS methods, this method is simpler, more accurate, more selective and more sensitive.

## 2. Experimental section

### 2.1 Instruments and reagents

#### 2.1.1 Instruments

We used a DXR Raman spectrometer (Thermo Co., Waltham) with a 633 nm laser, spectral range from 300 cm^−1^ to 1700 cm^−1^, laser power of 2.0 mW, integral time of 5 seconds and estimated resolution of 3.0 cm^−1^. We used an F-7000 fluorescence spectrophotometer with a voltage of 350 V, excitation slit = emission slit = 5 nm, emission filter = 1% T attenuator, and *λ*_ex_ − *λ*_em_ = Δ*λ* = 0 (Hitachi Co., Tokyo, Japan), an SK3300B sonic cleaning machine (Shanghai Kudos Ultrasonic Instrument Co., Ltd., Shanghai, China), a dual-beam UV-visible spectrophotometer with a model of TU-1901 (Beijing Puxi General Equipment Limited Co., Beijing, China), an HH-S2 constant temperature water bath kettle (Ointan Dadi Automatic Instrument Co., Changzhou, China), a high-speed freezing centrifuge (Shanghai Lu Xiangyi Centrifuge Instrument Co., Ltd., Shanghai, China), an electric thermostatic drying oven (Shanghai Jing Hong Laboratory Instrument Co., Ltd., Shanghai China), a vacuum-freeze dryer (Hangzhou Jtone Electronic Co., Ltd., Hangzhou, China), a digital display thermostatic water bath (Changzhou Guohua Electric Appliance Co., Ltd., Changzhou, China), a 79-1 magnetic heating stirrer (Jiangsu University Instrument Factory Co., Ltd., Jiangsu, China), a nanoparticle size and potential analyzer (Malvern Co., Malvern, England), a microwave digestion system (Shanghai Sineo Microwave Chemistry Technology Co., Ltd., Shanghai, China), a Fourier transform infrared FTIR spectrometer (Shanghai PerkinElmer Instruments Co., Ltd., Shanghai, China), an S-4800 field emission scanning electron microscope (Hitachi High-Technologies Corporation, Japan/Oxford Company, Oxford, U.K.), qualitative filter papers with an aperture of 20 μm (Hangzhou New StarFiber Co., Ltd., Hangzhou, China), and a Talos F200S field emission transmission electron microscope (Thermo Fisher Scientific, U.S.).

#### 2.1.2 Reagents

1,3,5-Benzene tricarboxaldehyde (Bt) (Jilin Chinese Academy of Sciences – Yanshen Technology Co., Ltd., Jilin, China), *p*-phenylenediamine (PD) (Sinopharm Chemical Reagent Co., Ltd., Shanghai, China), 1,4-dioxane (Xilong Scientific Co., Ltd., Shantou, China), 3 mol L^−1^ CH_3_COOH (Xilong Scientific Co., Ltd., Shantou, China), *N*,*N*-dimethylformamide (DMF) (Xilong Scientific Co., Ltd., Shantou, China), tetrahydrofuran (THF) (Xilong Scientific Co., Ltd., Shantou, China), 1% trisodium citrate (Xilong Scientific Co., Ltd., Shantou, China), sodium borohydride (Sinopharm Chemical Reagent Co., Ltd., Shanghai, China), 10 μmol L^−1^ Victoria blue 4R (VB4R) (Sinopharm Chemical Reagent Co., Ltd., Shanghai, China), 0.1 mol L^−1^ NaCl (Xilong Scientific Co., Ltd., Shantou, China), 0.01 mol L^−1^ HCl (Xilong Scientific Co., Ltd., Shantou, China), 0.1 mol L^−1^ sodium formate (Sinopharm Chemical Reagent Co., Ltd., Shanghai, China), 100 nmol L^−1^ Co(NO_3_)_2_·6H_2_O (Xilong Scientific Co., Ltd., Shantou, China), 1% HAuCl_4_ (Shanghai Macklin Biochemical Co., Ltd., Shanghai, China), and 0.1 μmol L^−1^ Apt with a sequence of 5′-3′ GGT AAT ACG ACT CAC TAA GGG AGA TAC CAG CTT ATT CAA TTT TAC AGA ACA CCA ACG TCG CTC GGG TAC TTC TTC ATC GAG ATA GTA AGT GCA ACT T (Shanghai Sangon Biotech Co., Ltd., Shanghai, China) were used. All reagents were of analytical grade, and the water was double distilled.

#### 2.1.3 Preparation of the nano-sol catalyst

##### 2.1.3.1 Synthesis of BtPD

Referring to the synthesis method of Wang *et al.*,^3^ we put Bt (48 mg) and 3 mL of 1,4-dioxane in a stoppered conical flask which was sonicated. Then 48 mg PD was added, and the solution turned brown. After slowly adding 0.6 mL of 3 mol L^−1^ acetic acid, it turned faint yellow. The solution was dried in a thermostatic blast oven at 120 °C for 3 days. Then, the product was successively washed with DMF (3 × 10 mL) and THF (3 × 10 mL). Finally, the product was vacuum-dried for 21 h to get a yellow powder (COF). 10 mL water and 1 mg products were added to a glass container and sonicated to obtain a 0.1 mg mL^−1^ BtPD solution.

##### 2.1.3.2 Synthesis of AuNPs

0.5 mL of 1% HAuCl_4_ solution and 3.5 mL of 1% trisodium citrate solution were mixed in a glass filled with 40 mL water. Then, 4.0 mL of 0.05% sodium borohydride solution was slowly added while stirring and stirring was continued for 10 min. Finally, the solution was diluted to 50 mL with water to obtain a 58 μg mL^−1^ AuNP solution.

##### 2.1.3.3 Synthesis of AuBtPD

100 μL of 0.1% HAuCl_4_ solution with 10 mL of 0.1 mg mL^−1^ BtPD were mixed in an Erlenmeyer flask. Then, 450 μL of 1.0% trisodium citrate solution was added into the flask which was placed on a magnetic stirrer. The rotation speed was adjusted to 200 rpm and the heating temperature to 60 °C. Finally, the solution, which was stirred for 15 min, was diluted to 15 mL with water to obtain a 0.067 mg mL^−1^ AuBtPD solution. To facilitate the calculation, we have referred to the concentration of AuBtPD as the concentration of BtPD.

### 2.2 Experimental method

In a 5.0 mL glass tube, 400 μL of 0.67 μg mL^−1^ AuBtPD solution and 240 μL of 0.1 μmol L^−1^ Apt solution were added. After 15 min, a certain concentration of Co(NO_3_)_2_ solution, 95 μL of 0.1% HAuCl_4_ solution, 100 μL of 0.01 mol L^−1^ HCl solution and 46 μL of 0.1 mol L^−1^ sodium formate (CHO_2_Na) solution were mixed, diluted to 1.5 mL, reacted in a 75 °C bath for 15 min, and rapidly cooled to room temperature with ice water. RRS spectra were recorded by synchronously scanning with a fluorescence spectrophotometer. The RRS intensity (*I*_370nm_) at 370 nm was measured, and the blank value (*I*_370nm_)_0_ was determined without adding the Co^2+^ solution. The value was calculated as Δ*I*_370nm_ = *I*_370nm_ − (*I*_370nm_)_0_. Then, 50 μL of 10 μmol L^−1^ VB4R was added in the above mixture. The SERS spectra were obtained *via* the Raman spectrometer. SERS intensity at the 1614 cm^−1^ Raman shift was measured, and the value was calculated as Δ*I*_1614cm^−1^_ = *I*_1614cm^−1^_ − (*I*_1614cm^−1^_)_0_.

## 3. Results and discussion

### 3.1 Principle

**Fig. 1:**
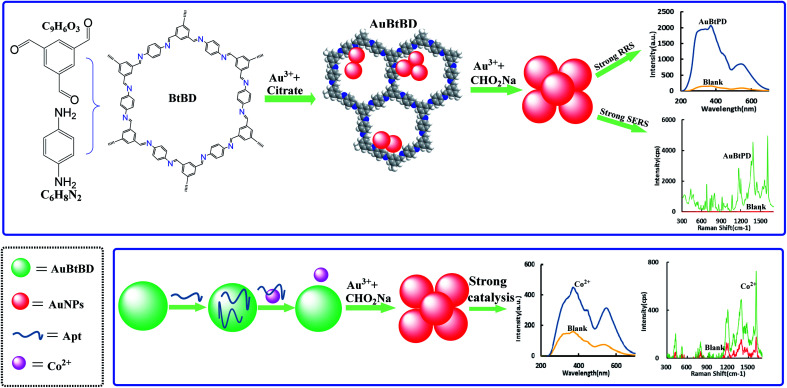
Gold nanosol SERS/RRS Apt assay of trace Co^2+^ by AuBtPD catalytic amplification.

We synthesized BtPD using Bt and PD as monomers and found that it has a catalytic effect on the reduction of HAuCl_4_ by CHO_2_Na to AuNPs, and AuBtPD had a stronger catalytic effect on the nanoreaction. The Apt can be adsorbed on the surface of AuBtPD to inhibit its catalytic action. When Co^2+^ was added, the Apt desorbed, and the catalytic performance of AuBtPD recovered. The generated AuNPs had strong RRS and SERS signals, which show a linear correlation with the concentration of Co^2+^ (Fig. 1), so as to achieve the purpose of detecting Co^2+^.

### 3.2 Material characterization

#### 3.2.1 Scanning electron microscopy (SEM), transmission electron microscopy (TEM), energy dispersive spectroscopy (EDS) and particle size distribution

**Fig. 2:**
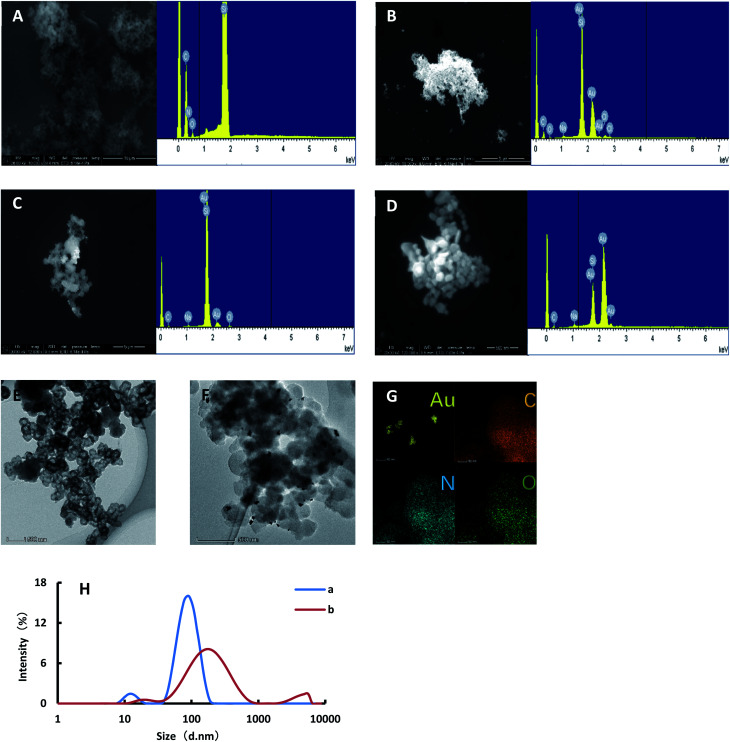
SEM, TEM, EDS and particle size distribution. (A): SEM of BtPD; (B): SEM of AuNPs; (C): SEM of the system for detecting Co^2+^ (0.064 mg mL^−1^ HAuCl_4_ + 0.67 mmol L^−1^ HCl + 3 mmol L^−1^ CHO_2_Na + 0.179 μg mL^−1^ AuBtPD + 16 nmol L^−1^ Apt + 0.167 nmol L^−1^ Co^2+^); (D): SEM of the system for detecting Co^2+^ (0.064 mg mL^−1^ HAuCl_4_ + 0.67 mmol L^−1^ HCl + 3 mmol L^−1^ CHO_2_Na + 0.179 μg mL^−1^ AuBtPD + 16 nmol L^−1^ Apt + 0.667 nmol L^−1^ Co^2+^); (E): TEM of BtPD; (F): TEM of AuBtPD; (G): element distribution of AuBtPD; (H): size distribution of the system for detecting Co^2+^ (a: as the conditions of C; b: as the conditions of D).

BtPD, AuNPs and AuBtPD were diluted with water, and 6 μL of solution was dropped onto a silicon wafer. The samples were placed into a scanning electron microscope for SEM and EDS after drying. As shown in Fig. 2A, BtPD was of different sizes and has a porous structure, and the EDS showed that only three elements C, N and O were found in BtPD. As shown in Fig. 2B, AuNPs did not disperse and had an average particle size of about 50 nm. According to the experimental method, the reaction solution was centrifuged at 10 000 rpm for 10 min, and then the solution was discarded. The precipitation was then dispersed by water. After centrifugation, 6 μL of solution was dropped onto the silicon wafer and dried. The samples were put into a scanning electron microscope for SEM and EDS. As shown in Fig. 2C, the Apt combined with Co^2+^ and separated from AuBtPD, and the catalytic performance of AuBtPD was recovered. A small amount of AuNPs was generated on AuBtPD. With the increasing concentration of Co^2+^, more and more gold nanoparticles were generated, and particle aggregation became more obvious (Fig. 2D). In addition, we finished the TEM and EDS of AuBtPD. The steps are as follows: AuBtPD was diluted with ethanol, then added to the copper net and dried, and the sample was put into the transmission electron microscope for TEM and element distribution. As shown in Fig. 2E and F, the morphology of AuBtPD did not change. The results showed that gold nanoparticles were well attached and dispersed on the surface of the COF, and the average particle size of AuNPs was 10 nm. The element distribution of AuBtPD is shown in Fig. 2G, which contains four elements: Au, C, N and O. The AuNPs generated by the detection system were analyzed by using a nanoparticle size analyzer. When the concentration of Co^2+^ was low, the average particle size of the generated AuNPs was 106 nm. With the increasing concentration of Co^2+^, the catalytic performance of AuBtPD recovered; the generation rate of gold nanoparticles accelerated and the particle size increased, with an average of 150 nm (Fig. 2H).

#### 3.2.2 Molecular spectra of the COF

**Fig. 3:**
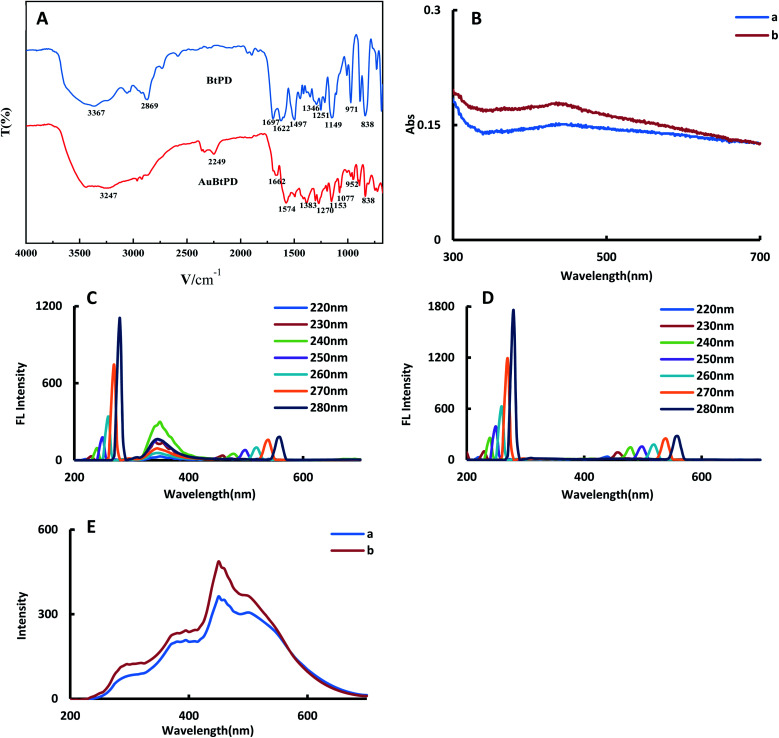
Molecular spectrum (A): Fourier transform infrared spectra of BtPD and AuBtPD; (B): absorption spectra of BtPD and AuBtPD; (C): FL spectra of BtPD; (D): FL spectra of AuBtPD; (E): RRS spectra of BtPD and AuBtPD (a: 0.1 mg mL^−1^ BtPD; b: 0.1 mg mL^−1^ AuBtPD).

BtPD and AuBtPD powders were scanned using a Fourier transform infrared spectrometer (Fig. 3A). The absorption peak of BtPD at 3367 cm^−1^ indicates that there was a free O–H stretching vibration. The absorption peak at 2869 cm^−1^ indicated the stretching vibration of C–H of the aldehyde group. The absorption peak at 1697 cm^−1^ may be the C=N stretching vibration of imines. The absorption peak at 1622 cm^−1^ was caused by the skeleton vibration of the benzene ring. The absorption peak at 1497 cm^−1^ may be the stretching vibration of the aromatic compound C=C. The absorption peak at 1346 cm^−1^ may be the C–N stretching vibration. The absorption peaks at 1251 cm^−1^ and 1149 cm^−1^ were the C–C structural vibration. The absorption peak at 971 cm^−1^ was the C–H structure with the aldehyde group. The absorption peak at 838 cm^−1^ may be the –NH_2_ deformation vibration. Due to doping Au, the structure of the COF changed, and the peak value of the functional group showed an obvious blue shift (Table S1†).

For the convenience of comparing the differences before and after doping gold nanoparticles, we investigated the absorption spectra, fluorescence spectra (FL) and RRS spectra of BtPD and AuBtPD at the same concentration. In the absorption spectra, AuBtPD has an obvious surface plasma resonance absorption peak at 430 nm compared with BtPD, which belongs to the absorption peak of gold nanoparticles (Fig. 3B). The fluorescence properties of BtPD and AuBtPD with different excitation wavelengths were investigated. BtPD has a significant fluorescence signal due to rigid conjugated π bonds of benzene rings (Fig. 3C). In the range of the excitation wavelength of 220–280 nm, BtPD showed an obvious fluorescence signal at 345 nm, and it was most obvious when excited at 240 nm. However the fluorescence signal of AuBtPD was seriously quenched due to energy transfer between the COF and the metal in the process of doping gold nanoparticles with BtPD (Fig. 3D).^34^ In the RRS spectra, the peak generated by AuBtPD was more obvious than the peak generated by BtPD at 450 nm (Fig. 3F). The stability of BtPD and AuBtPD sol was investigated using RRS spectra. The stability of 0.1 mg mL^−1^ AuBtPD and 0.1 mg mL^−1^ BtPD sol was stabilized within 15 days, and the RRS signal did not change significantly (Fig. S3†). In 0.1 mg mL^−1^ BtPD and 0.1 mg mL^−1^ AuBtPD sol, 1–18 mmol L^−1^ NaCl solution was added, respectively, and the RRS signal remained unchanged, indicating that it was not aggregated by salt.

### 3.3 RRS spectra of the nanocatalytic system

**Fig. 4:**
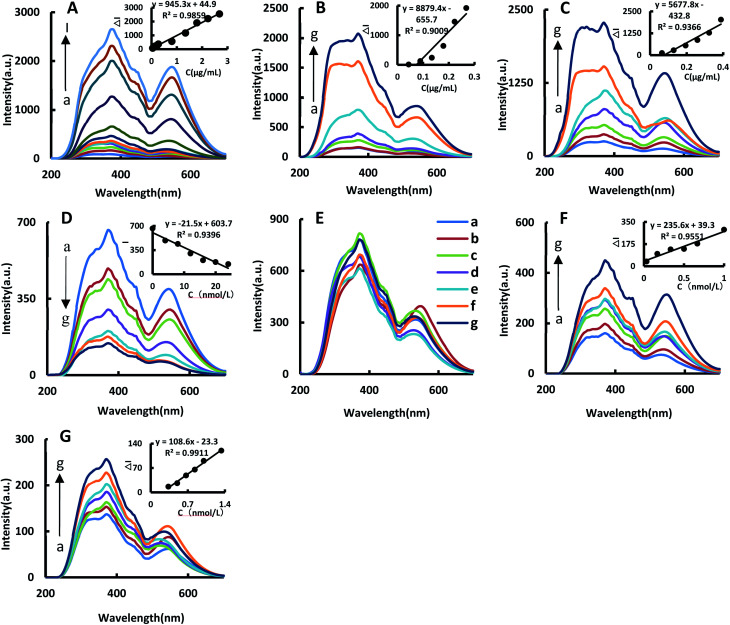
RRS spectra of the catalytic system. (A): 0.067 mg mL^−1^ HAuCl_4_ + 0.67 mmol L^−1^ HCl + 3 mmol L^−1^ CHO_2_Na, (a–l) were 0, 0.044, 0.089, 0.134, 0.179, 0.223, 0.268, 0.804, 1.34, 1.79, 2.23 and 2.68 μg mL^−1^ BtPD, respectively. (B): 0.067 mg mL^−1^ HAuCl_4_ + 0.67 mmol L^−1^ HCl + 3 mmol L^−1^ CHO_2_Na, (a–g) represent 0, 0.044, 0.089, 0.134, 0.179, 0.223 and 0.268 μg mL^−1^ AuBtPD, respectively. (C): 0.067 mg mL^−1^ HAuCl_4_ + 0.67 mmol L^−1^ HCl + 3 mmol L^−1^ CHO_2_Na, (a–g) represent 0, 0.065, 0.129, 0.194, 0.259, 0.323 and 0.388 μg mL^−1^ AuNPs, respectively. (D): 0.067 mg mL^−1^ HAuCl_4_ + 0.67 mmol L^−1^ HCl + 3 mmol L^−1^ CHO_2_Na + 0.145 μg mL^−1^ AuNPs, (a–g) represent 0, 66.67, 133.3, 6.67, 13.3, 0.67 and 1.33 nmol L^−1^ Apt, respectively. (E): 0.067 mg mL^−1^ HAuCl_4_ + 0.67 mmol L^−1^ HCl + 3 mmol L^−1^ CHO_2_Na + 0.179 μg mL^−1^ AuBtPD, (a–g) represent 0, 4, 8, 12, 16, 20 and 24 nmol L^−1^ Apt, respectively. (F): 0.064 mg mL^−1^ HAuCl_4_ + 0.67 mmol L^−1^ HCl + 3 mmol L^−1^ CHO_2_Na + 0.179 μg mL^−1^ AuBtPD + 16 nmol L^−1^ Apt, (a–g) represent 0, 0.033, 0.167, 0.333, 0.5, 0.667 and 1 nmol L^−1^ Co^2+^, respectively. (G): 0.064 mg mL^−1^ HAuCl_4_ + 0.67 mmol L^−1^ HCl + 3 mmol L^−1^ CHO_2_Na + 0.179 μg mL^−1^ BtPD + 16 nmol L^−1^ Apt, (a–g) represent 0, 0.333, 0.5, 0.667, 0.833, 1 and 1.333 nmol L^−1^ Co^2+^, respectively.

The catalytic effects of BtPD, AuBtPD and AuNPs on the reaction were investigated. The system has three spectral peaks at 370 nm, 450 nm and 530 nm. In a certain concentration range, the RRS signal increased linearly with the concentration of BtPD, AuBtPD and AuNPs increasing; the RRS in the 370 nm peak was the largest in strength due to changes in the relationship between Δ*I*_370nm_ and the catalyst concentration to make a working curve to compare the catalytic performance between BtPD, AuBtPD and AuNPs (Fig. 4A–C). The linear summary of the catalytic effects of the three catalysts on the reaction system is shown in Table 1. It can be seen from the table that BtPD, AuBtPD and AuNPs all had catalytic effects on the system of CHO_2_Na reducing auric acid chloride, and the catalytic performance of BtPD can be further improved by loading gold nanoparticles. For the convenience of comparing AuBtPD and AuNPs catalytic performance difference of the system, we compared the concentration of gold nanoparticles in the two catalysts. Although AuNPs have a strong catalytic effect on the reaction system, AuNPs that used alone were at a concentration about 3.8 times higher than that loaded on BtPD, but their catalytic performance was not as strong as AuBtPD. The reason was that the particle size of AuNPs in the two materials was different, and there may be a large amount of particle aggregation in AuNPs, which affected the catalytic performance. Both BtPD and AuNPs could catalyze the HAuCl_4_–CHO_2_Na system. The synergistic effect of BtPD and AuNPs can greatly enhance the catalytic performance; BtPD also played a good role in dispersing AuNPs (Fig. 2D) and improved the utilization rate. As a result, the catalytic efficiency of BtPD loaded AuNPs was improved.

**Table 1:** Catalytic effects of nanoparticles on the reaction of HAuCl_4_–CHO_2_Na

Nanoparticle	Linear range	Regression equation	*R* ^2^
BtPD	0.044–2.68 μg mL^−1^	Δ*I*_370nm_ = 945.3C + 44.9	0.9859
AuBtPD	0.044–0.268 μg mL^−1^	Δ*I*_370nm_ = 8879.4C − 655.7	0.9009
AuBtPD^a^	0.017–0.102 μg mL^−1^	Δ*I*_370nm_ = 23409C − 661.8	0.9027
AuNPs	0.065–0.388 μg mL^−1^	Δ*I*_370nm_ = 5677.8C − 432.8	0.9366

a Calculated in gold nanoparticle (AuNP) concentration.

Due to the presence of a large number of stabilizers such as citrate on the surface of AuNPs, the adhesion of the Apt to AuNPs could not inhibit their catalytic performance, as shown in Fig. 4E, so it cannot be used to detect Co^2+^.

In the Apt–AuBtPD–HAuCl_4_–CHO_2_Na–HCl system, the Apt would attach to AuBtPD and form the Apt–AuBtPD complex, which could inhibit the catalytic reaction of the system (Fig. 4D). The concentration of the Apt solution in the range of 4–24 nmol L^−1^ showed a good linear relationship with the intensity at 370 nm of RRS. Both AuBtPD and BtPD detection systems all exhibit two RRS peaks at 370 nm and 530 nm. The former peak was stronger than the latter and was selected for the assay of Co^2+^. In this detection system, Co^2+^ specifically bound with the corresponding Apt and AuBtPD was released. As the concentration of Co^2+^ increased, the RRS signal at 370 nm gradually increased. The relationship between Δ*I*_370nm_ and the concentration of Co^2+^ was selected to draw a working curve (Fig. 4F). The RRS signal changes of the detection system catalyzed by BtPD with the same concentration as AuBtPD were listed for comparison (Fig. 4G). The catalytic performance of AuBtPD was stronger than that of BtPD, so the former was chosen for use.

### 3.4 Absorption spectra of the catalytic system

In the BtPD catalytic system, the solution gradually turned pale blue. It generated a characteristic peak at 550 nm and the absorbance gradually increased as the catalyst concentration increased (Fig. S1A†). In the catalytic system of AuBtPD and AuNPs, with the increase of catalyst concentration, the solution gradually turned light wine red. There is a characteristic peak at 530 nm and the absorbance gradually increased as the catalyst concentration increased (Fig. S1B†). Compared with that of BtPD (Fig. S1C†), the signal of the reaction system catalyzed by AuBtPD was more obvious, indicating that the catalytic performance of BtPD was significantly improved after doping AuNPs. In this detection system, with the increase of Co^2+^ concentration within a certain range, because the released AuBtPD of the reaction system gradually increased, the number of gold nanoparticles generated increased, and the absorbance at 570 nm increased significantly (Fig. S1D†). Interestingly, we found that in the AuBtPD and AuNPs catalytic systems, with the increase of the concentration of the two catalysts, the absorption peak produced an obvious blue shift. In general, the absorption peak is red shifted with the increase of nanoparticle size. We explained this phenomenon according to the research by S. L. Smitha *et al.* The generated nanoparticle absorption spectrum is affected by the surface plasmon resonance, depends on the particle size, shape, state of aggregation and the surrounding dielectric medium, and the excess reductant during the preparation of gold nanoparticles can lead to a blue shift of the absorption peak.^35^ In this way, in order to generate gold nanoparticles completely, we added excessive reducing agents in the preparation of these two materials, causing the blue shift. Therefore, as seen in Fig. S2h,† the amount of the reducing agent was optimized to reduce the influence of the reducing agent. Another reason is that in the catalytic system of AuBtPD, the particle size of gold nanoparticles generated is different. The small gold nanoparticles will adhere to and gather on AuBtPD, which affects the surface plasmon resonance of larger AuBtPD. However, the large gold nanoparticles will fall off from AuBtPD. The aggregation disappears, resulting in the blue shift of the absorption peak. However, in the BtPD catalytic system and the AuBtPD detection of the Co^2+^ system, most of the generated small gold nanoparticles are attached to BtPD and AuBtPD (Fig. 2B), resulting in no shift of the absorption peak.

### 3.5 SERS spectra of the catalytic system

**Fig. 5:**
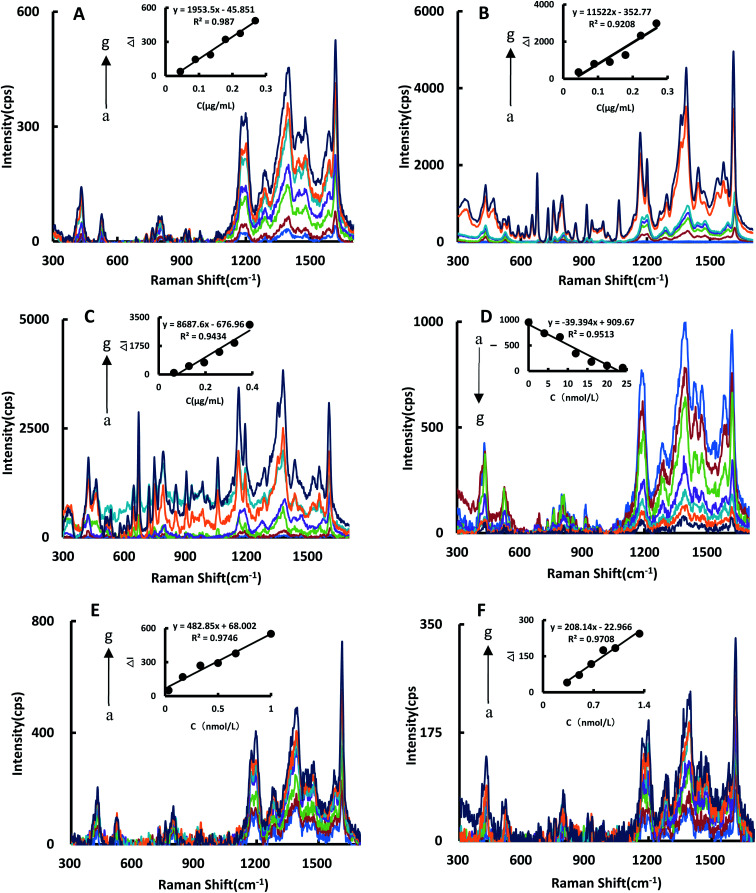
SERS spectra (A): 0.067 mg mL^−1^ HAuCl_4_ + 0.67 mmol L^−1^ HCl + 3 mmol L^−1^ CHO_2_Na, (a–g) represent 0, 0.044, 0.089, 0.134, 0.179, 0.223 and 0.268 μg mL^−1^ BtPD, respectively. (B): 0.067 mg mL^−1^ HAuCl_4_ + 0.67 mmol L^−1^ HCl + 3 mmol L^−1^ CHO_2_Na, (a–g) represent 0, 0.044, 0.089, 0.134, 0.179, 0.223 and 0.268 μg mL^−1^ AuBtPD, respectively. (C): 0.067 mg mL^−1^ HAuCl_4_ + 0.67 mmol L^−1^ HCl + 3 mmol L^−1^ CHO_2_Na, (a–g) represent 0, 0.065, 0.129, 0.194, 0.259, 0.323 and 0.388 μg mL^−1^ AuNPs, respectively. (D): 0.067 mg mL^−1^ HAuCl_4_ + 0.67 mmol L^−1^ HCl + 3 mmol L^−1^ CHO_2_Na + 0.179 μg mL^−1^ AuBtPD, (a–g) represent 0, 4, 8, 12, 16, 20 and 24 nmol L^−1^ Apt, respectively. (E): 0.064 mg mL^−1^ HAuCl_4_ + 0.67 mmol L^−1^ HCl + 3 mmol L^−1^ CHO_2_Na + 0.179 μg mL^−1^ AuBtPD + 16 nmol L^−1^ Apt, (a–g) represent 0, 0.033, 0.167, 0.333, 0.5, 0.667 and 1 nmol L^−1^ Co^2+^, respectively. (F): 0.064 mg mL^−1^ HAuCl_4_ + 0.67 mmol L^−1^ HCl + 3 mmol L^−1^ CHO_2_Na + 0.179 μg mL^−1^ BtPD + 16 nmol L^−1^ Apt, (a–g) represent 0, 0.333, 0.5, 0.667, 0.833, 1 and 1.333 nmol L^−1^ Co^2+^, respectively.

The SERS spectra of the reaction system catalyzed by BtPD and AuBtPD were studied (Fig. 5A and B), and there were significant SERS peaks at 1169 cm^−1^, 1405 cm^−1^, 1618 cm^−1^ and so on. With the increase of the two catalysts’ concentrations, the SERS signals of the system were significantly enhanced, among which the signal changes at 1618 cm^−1^ were obvious with good linearity. Therefore, the SERS intensity at 1618 cm^−1^ was chosen to use, and the results show that the catalytic effect of AuBtPD was far greater than that of BtPD. AuNPs that used alone in the reaction system were at a concentration about 3.8 times higher than that loaded on BtPD, but their catalytic performance was still weaker than that of AuBtPD (Fig. 5C). The effect of the aptamer on AuBtPD is shown in Fig. 5D. The aptamer and AuBtPD formed the Apt–AuBtPD complex, which inhibited the catalytic performance of AuBtPD. In the detection system catalyzed by AuBtPD and regulated by the Apt, along with the increase of Co^2+^ concentration, the SERS intensity was gradually increased, and the signal was obvious at 1614 cm^−1^. The relationship between Δ*I*_1614cm^−1^_ and Co^2+^ concentration can be used to draw a working curve (Fig. 5E). By comparing the SERS peak of the catalytic system and detection system, we found that the peak of the detection system had a blue shift. According to the research of Sung *et al.*,^36^ gold nanoparticles would interact with C–N, resulting in a slight blue shift. There was evidence that the Apt used in this experiment would adsorb on AuNPs.^42^ Therefore, in the detection system, we deduced that a small amount of Apt that combined with CO^2+^ would adsorb on the surface of the generated gold nanoparticles and cause the blue shift. However, it had no effect on the detection of CO^2+^. Similarly, the detection system catalyzed by BtPD was given as a comparison (Fig. 5F). It can be seen that due to the stronger catalytic performance of AuBtPD, the sensitivity and signal intensity of the AuBtPD system were significantly improved. The concentration of Co^2+^ shows a good linear relationship with Δ*I*_1614cm^−1^_ in the range of 0.033–1 nmol L^−1^, and the linear correlation coefficient was 0.9746. The generated gold nanoparticles were used as the substrate for SERS detection, but due to lack of surface protectors, they would gather over time. Therefore, the stability of the generated gold nanoparticles was very important. We tested the gold nanoparticles generated in the detection system of 5 batches (Fig. S4†). The results showed that the gold nanoparticles generated in the system basically had no effect on the SERS detection within 1 hour, and the relative error between different batches was kept within 10%.

### 3.6 Optimization of experimental conditions

According to the experimental method, the reaction conditions were optimized (Fig. S2†). The concentration of HAuCl_4_ in the system was optimized, and it was found that at 0.064 mg mL^−1^, Δ*I*_370nm_ reached its maximum value. The value of Δ*I*_370nm_ reached its maximum when the concentration of CHO_2_Na was 3 mmol L^−1^. When HCl concentration was 0.8 mmol L^−1^, Δ*I*_370nm_ reached its maximum value. The value of Δ*I*_370nm_ reached its maximum when the concentration of AuBtPD was 0.179 μg mL^−1^. The concentration of the aptamer was optimized, and at 16 nmol L^−1^, Δ*I*_370nm_ reached its maximum value. The water bath temperature and time of the reaction system were optimized. When the bath temperature was 75 °C and the bath time was 25 min, Δ*I*_370nm_ reached the maximum value. The amount of trisodium citrate (10 mg mL^−1^) was optimized for the preparation of AuBtPD, and at the added volume of 450 μL, Δ*I*_370nm_ reached the maximum value.

### 3.7 Working curve

By comparing the catalytic performance of the two materials on the system, it can be seen that the catalytic performance of AuBtPD is better, and the working curve of AuBtPD applied to the analysis and determination of Co^2+^ is shown in Table 2. The working curve of RRS is Δ*I*_370nm_ = 235.6C + 39.3 and the correlation coefficient is 0.9551, with a linear range of 0.033–1 nmol L^−1^ and a LOD of 0.024 nmol L^−1^. The working curve of SERS is Δ*I*_1618cm^−1^_ = 482.8C + 68.0, with the correlation coefficient of 0.9746. The linear range is 0.033–1 nmol L^−1^ and the LOD is 0.020 nmol L^−1^. The sensitivity of the BtPD detection system is lower than that of AuBtPD. By comparing the slope of the working curve between the RRS method and SERS method, the sensitivity of SERS determination is higher than that of RRS. Compared with the reported methods for the determination of Co^2+^ (Table 3), this method is simple, sensitive and selective. The material of this method is easy to prepare, and the analysis process is fast, and the dual-mode method ensures the accuracy of this analysis. It is one of the most sensitive methods for the determination of Co^2+^.

**Table 2:** Analytical characteristics of Apt-regulated nanocatalysis-SERS/RRS for Co^2+^

Catalytic system	Method	Linear range (nmol L^−1^)	Regression equation	*R* ^2^	LOD (nmol L^−1^)
AuBtPD	SERS	0.033–1	Δ*I*_1614cm^−1^_ = 482.8C + 68.0	0.9746	0.02
BtPD	SERS	0.33–1.3	Δ*I*_1613cm^−1^_ = 208.14C − 23.0	0.9708	0.2
AuBtPD	RRS	0.033–1	Δ*I*_370nm_ = 235.6C + 39.3	0.9551	0.02
BtPD	RRS	0.33–1.3	Δ*I*_370nm_ = 108.6C − 23.3	0.9911	0.3

**Table 3:** Comparison of reported analytical methods for Co^2+^^a^^b^

Method	Principle	Linear range	LOD	Ref.
RRS	The mixed solution of Co^2+^ with thiocyanate and neutral red (NR) generated RRS intensity in the presence of PVA-124, and the RRS intensity was proportional to the concentration of cobalt	0–0.48 μg mL^−1^	4.4 × 10^−5^ μg mL^−1^	33
SERS	Preparing silver nanoparticles functionalized with dithiocarbamate salt, this material had a high affinity for CO^2+^ and CO^2+^ can make the SERS spectrum of the material change	0–5.9 ppb	60 ppt	31
FL	A gold nanocluster probe with a fluorescence signal was prepared using methionine as the reducing agent and protectant, and selectively quenched by Co^2+^	0.001–2 μmol L^−1^	0.42 nmol L^−1^	37
Electrochemical	A composite electrochemical sensor was prepared by an *in situ* chemical polymerization method. This sensor has higher responsiveness to Co^2+^ than other ions and can be used to detect Co^2+^	0.1–0.1 mmol L^−1^	94.67 pmol L^−1^	38
SPR	A gold active layer modified by chitosan-GO/CdS quantum dots was prepared. It can be combined with Co^2+^, resulting in changes in the refractive index of the sensing layer, and Co^2+^ can be detected by the SPR signal	1–10 ppm	0.01 ppm	39
ECL	A gold electrode was modified by a nanocomposite, and then preparing MIP by electropolymerization. Co^2+^ can be selectively recognized by BSA in MIP, and the BSA–Co^2+^ complex quenched the ECL signal. Therefore, Co^2+^ can be detected by double recognition	1–100 nmol L^−1^	0.307 nmol L^−1^	40
Grey level/UV-vis spectra/DLS	Preparing a nanoprobe by using thioglycolic acid-capped ZnSe quantum dots. When Co^2+^ was added, the color changed. Co^2+^ was detected *via* the measurement of the grey level, UV-vis spectra and DLS	5–1000 mg L^−1^	2.6 mg L^−1^	41
		0.5–50 mg L^−1^	0.14 mg L^−1^	
		0.1–10 mg L^−1^	3.0 μg L^−1^	
CL	Preparing the Ag–Au nanoalloy to catalyze the oxidation of luminol by H_2_O_2_ to produce chemiluminescence. The Apt can be combined with the material to regulate the catalysis. When Co^2+^ was added, the catalysis was released, and Co^2+^ can be detected through the CL signal	0.01–10 μg L^−1^	0.001 μg L^−1^	42
SERS/RRS	The gold-doped COF has a strong catalysis in the Au^3+^–CHO_2_Na system, and the Apt could inhibit its activity. APT and Co^2+^ formed APT–Co complex after adding Co^2+^, and the catalysis of gold-doped COF was recovered. The generated AuNPs had strong RRS and SERS signals	0.033–1 nmol L^−1^	RRS 0.02 nmol L^−1^	This method
			SERS 0.02 nmol L^−1^	

a FL: fluorescence; SPR: surface plasmon resonance; ECL: electro-chemiluminescence; DLS: dynamic light scattering.

b CL: chemiluminescence; SERS: surface enhanced Raman scattering; RRS: resonance Rayleigh scattering.

### 3.8 Influence of interfering ions

According to the experiment method, the effect of coexisting ions on the determination of 0.667 nmol L^−1^ Co^2+^ in the SERS/RRS system was investigated. The results showed that 10–1000 times the concentration of common ions have no effect on the measurement of Co^2+^ with the relative error within 10% (Tables S2 and S3†). It showed that this method has good selectivity.

### 3.9 Analysis of the sample

The samples of tap water, river water and waste water were analysed. River water was from the Xiangsi River near by Guangxi Normal University in Guilin. Tap water was from Bokang building in Guangxi Normal University in Guilin. Waste water from a certain auto parts manufacturing factory in Liuzhou city was collected. The samples were filtered to remove suspended solids and digested by microwave. Diluting them to a certain multiple and then dividing each sample into five parts, these were analyzed with this method. 0.50 nmol L^−1^ Co^2+^ was added for the recovery experiment, and the determination results are shown in Tables S4 and S5.† The recovery rate of the RRS method was between 91–106% and relative standard deviation was between 2.14–8.39%; the recovery rate of the SERS method was between 92–101% and the relative standard deviation was between 4.06–9.22%.

## 4. Conclusions

In this paper, AuBtPD nanosol with high catalytic activity and high stability was synthesized by means of a polycondensation–reduction procedure using Bt and PD, auric chloride and citrate as precursors. It was characterized by SEM, TEM, IR spectroscopy and using a particle size analyzer. We studied the catalytic performance before and after it was loaded with nanogold by analysing the RSS/SERS/Abs spectra, and it was found that stable AuBtPD nanosol can significantly improve the catalytic performance on the indicator nanoreaction. Although the AuNPs that used alone have a catalytic role on the indicator nanoreaction, they were not scattered enough, and the Apt has no inhibitory effect on them. This demonstrated that the COF-loaded AuNPs solved the problem of instability of AuNPs. Thus, a new dual-mode RRS/SERS Apt method for the determination of Co^2+^ in water was developed by using the strategy of nanocatalytic amplification. This method is simple, sensitive, accurate and selective, and can be applied to the determination of Co^2+^ in real samples.

## Conflicts of interest

There are no conflicts to declare.

## Supplementary Material

NA-003-D1NA00208B-s001
