# Supplementary material for: A nanosol SERS/RRS aptamer assay of trace cobalt(ii) by covalent organic framework BtPD-loaded nanogold catalytic amplification
Source: Nanoscale Adv. 2021 May 5;3(13):3846–59. doi: 10.1039/d1na00208b (PMC9417635; doi:10.1039/d1na00208b)
Supplement: NA-003-D1NA00208B-s001 [file NA-003-D1NA00208B-s001.pdf]

Electronic Supplementary Material (ESI) for Nanoscale Advances.
This journal is © The Royal Society of Chemistry 2021

Nanosol SERS/RRS aptamer assay of trace cobalt (II) by covalent organic framework BtPD-loaded nanogold catalytic amplification

Guiqing Wen, Yang Xiao, Shuxin Chen, Xinghui Zhang, Zhiliang Jiang*
Key Laboratory of Ecology of Rare and Endangered Species and Environmental Protection (Guangxi Normal University), Ministry of Education, Guangxi, Key Laboratory of Environmental Pollution Control Theory and Technology, Guilin 541004, China.

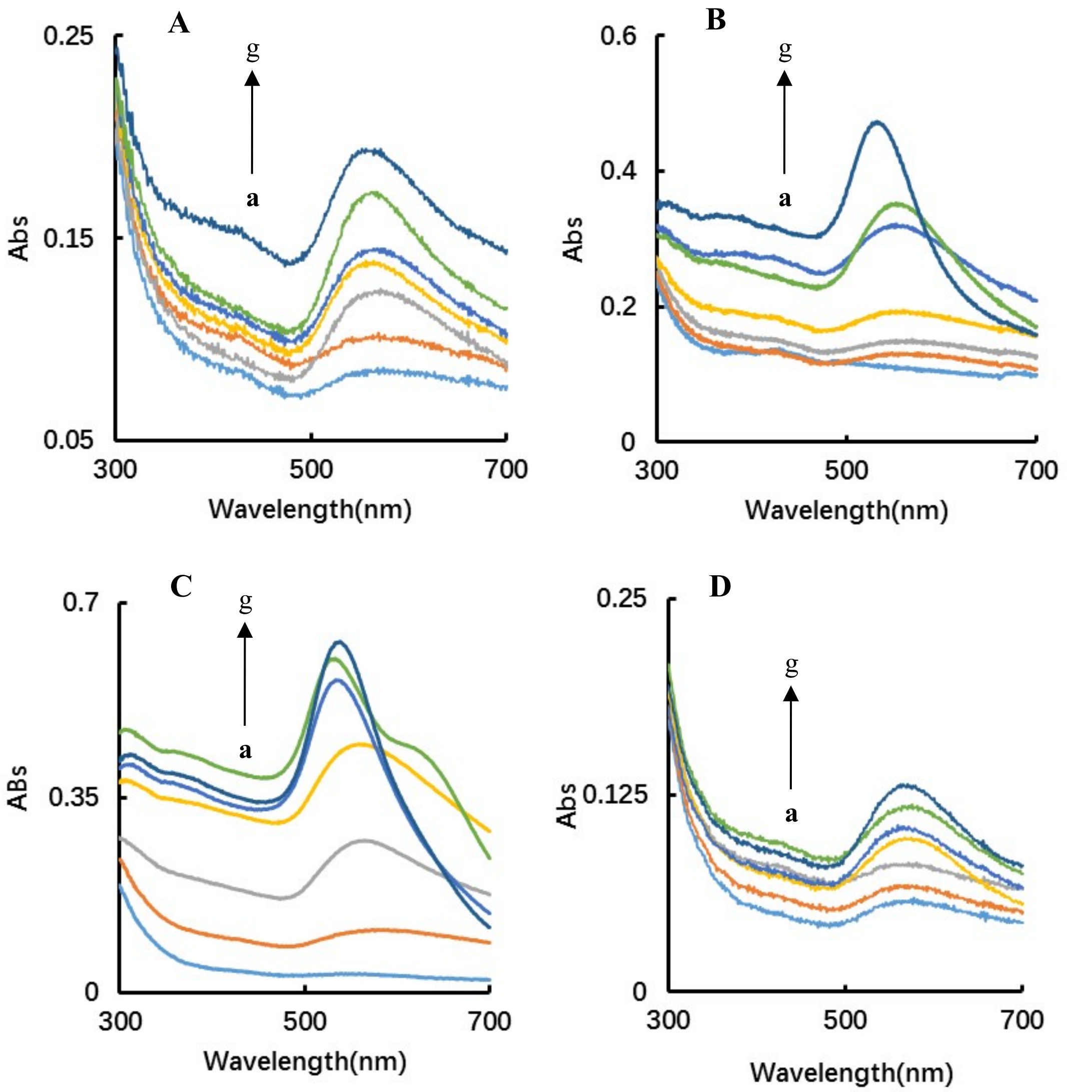

Fig. S1 Absorption spectra of the catalytic system

A: a: 0.067 mg/mL $HAuCl_4$+0.67 mmol/L HCl +3 mmol/L CHO_2Na; b-g: a+0.044, 0.089, 0.134, 0.179, 0.223, 0.268 μg/mL BtPD.

B: a: 0.067 mg/mL $HAuCl_4$+0.67 mmol/L HCl+3 mmol/L CHO_2Na; b-g: a+0.044, 0.089,

0.134, 0.179, 0.223, 0.268 μg/mL AuBtPD.

C: 0.067 mg/mL $HAuCl_4$+0.67 mmol/L HCl + 3 mmol/L CHO_2Na; b-g: a+0.065, 0.129, 0.194, 0.259, 0.323, 0.388 μg/mL AuNPs.

D: a: 0.064 mg/mL $HAuCl_4$+0.67 mmol/L HCl + 3 mmol/L CHO_2Na + 0.179 μg/mL AuBtPD + 16 nmol/L Apt_{Co}; b-g: a+0.033, 0.167, 0.333, 0.5, 0.667, 1 nmol/L Co^{2+}.

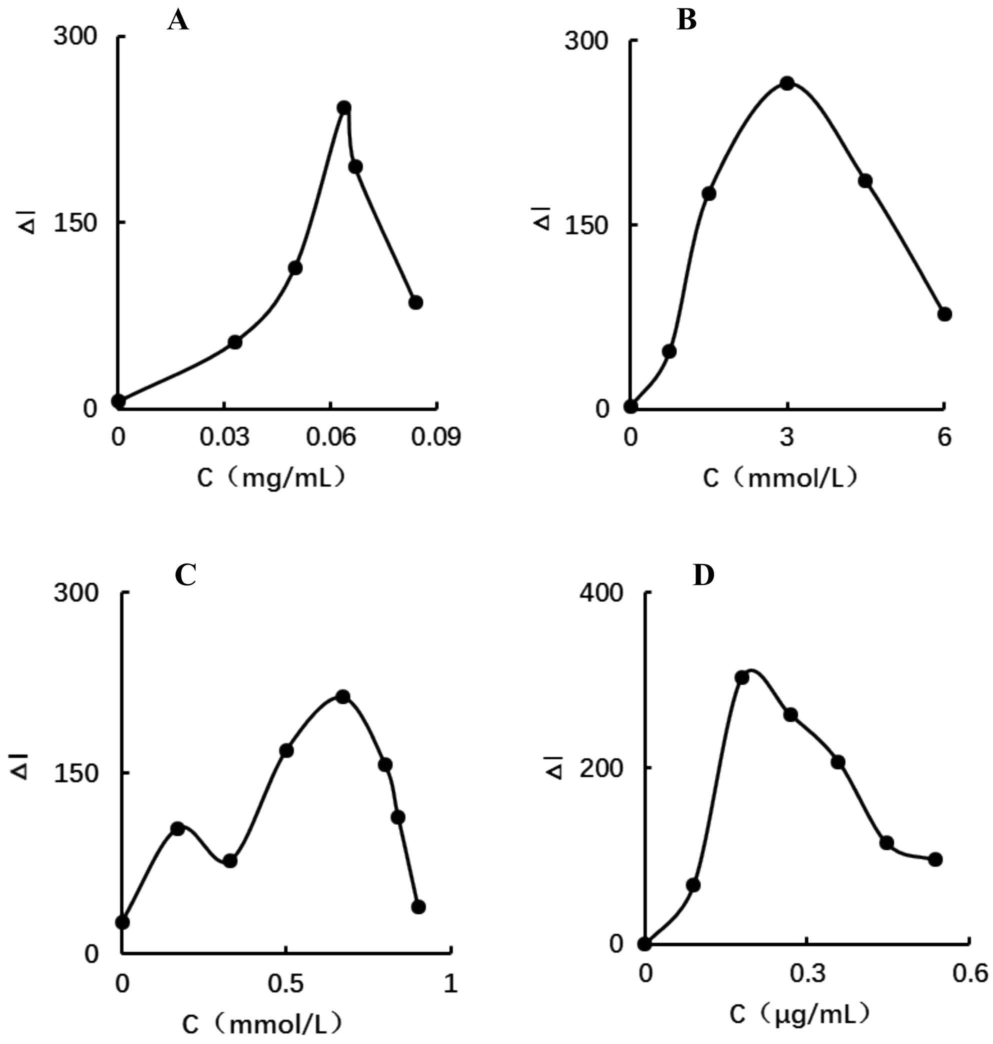

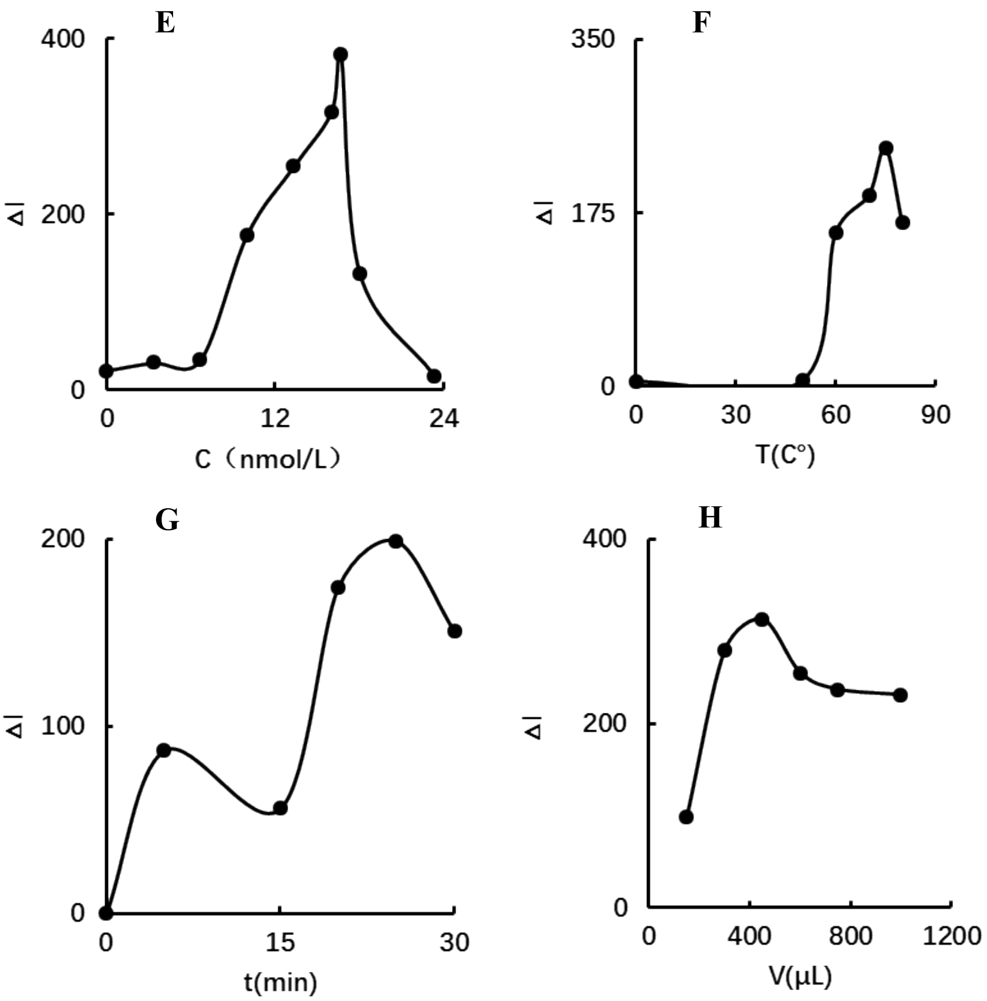

Fig. S2 Conditions optimization

A: $HAuCl_4$ concentration: $HAuCl_4$ + 0.67 mmol/L HCl + 3 mmol/L CHO_2Na + 0.179 μg/mL AuBtPD + 16 nmol/L Apt_{Co} + 0.667 nmol/L Co^{2+}.

B: Influence of CHO_2Na concentration: CHO_2Na + 0.064 mg/mL $HAuCl_4$ + 0.67 mmol/L HCl + 0.179 μg/mL AuBtPD + 16 nmol/L Apt_{Co} + 0.667 nmol/L Co^{2+}.

C: Influence of HCl concentration: HCl + 0.064mg/mL $HAuCl_4$ + 3 mmol/L CHO_2Na + 0.179 μg/mL AuBtPD + 16 nmol/L Apt_{Co} + 0.667 nmol/L Co^{2+}.

D: Influence of AuBtPD concentration: AuBtPD + 0.064 mg/mL $HAuCl_4$ + 0.67 mmol/L HCl + 3 mmol/L CHO_2Na + 16 nmol/L Apt_{Co} + 0.667 nmol/L Co^{2+}.

E: Influence of Apt_{Co} concentration: atp_{co} + 0.064 mg/mL $HAuCl_4$ + 0.67 mmol/L HCl + 3 mmol/L CHO_2Na + 0.179 μg/mL AuBtPD + 0.667 nmol/L Co^{2+}.

F: Influence of water bath temperature: 0.064 mg/mL $HAuCl_4$ + 0.67 mmol/L HCl + 3 mmol/L CHO_2Na + 0.179 μg/mL AuBtPD + 16 nmol/L Apt_{Co} + 0.667 nmol/L Co^{2+}.

G: Influence of water bath time: 0.064 mg/mL $HAuCl_4$ + 0.67 mmol/L HCl + 3 mmol/L CHO_2Na + 0.179 μg/mL AuBtPD + 16 nmol/L Apt_{Co} + 0.667 nmol/L Co^{2+}.

H: Influence of the added volume of trisodium citrate: 0.064 mg/mL $HAuCl_4$ + 0.67 mmol/L HCl + 3 mmol/L CHO_2Na + 0.179 μg/mL AuBtPD + 16 nmol/L Apt_{Co} + 0.667 nmol/L Co^{2+}.

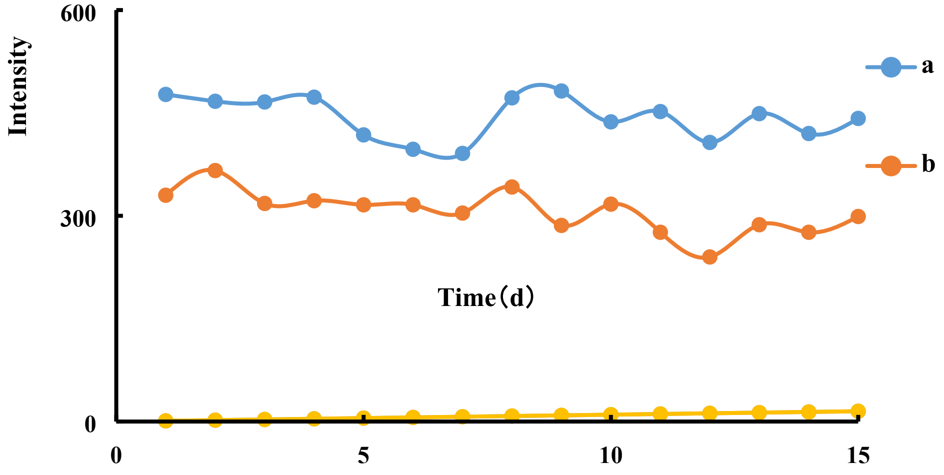

Fig. S3 RRS intensity of COF

a: 0.1 mg/mL AuBtPD ; **b:** 0.1 mg/mL BtPD.

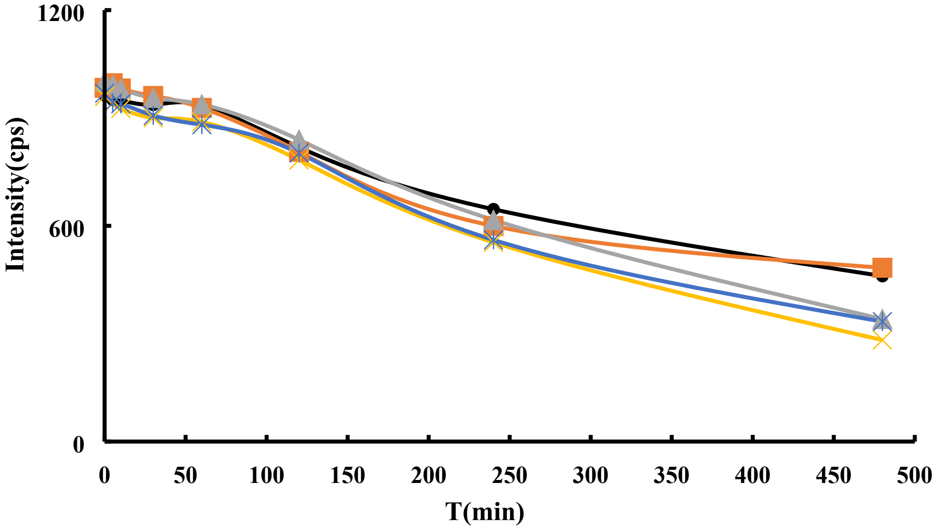

Fig. S4 SERS Intensity of different batches

0.064 mg/mL HAuCl4+ 0.67 mmol/L HCl + 3 mmol/L CHO_2Na + 0.179 μg/mL AuBtPD + 16nmol/L Apt + 1 nmol/L Co^{2+}.

Table S1 FTIR peaks and the corresponding functional groups

	BtPD	AuBtPD
functional group	IR peak(cm^{-1})	IR peak(cm^{-1})
O−H	3367	3247
C＝N	1697	1662
benzene ring	1622	1574
C＝C	1497	1383
C−N	1346	1270
C−C	1251、1149	1077
C−H	971	952
$-NH_2$	838	836

Table S2 Influence of coexisting substances on SERS system

Coexisting substances	Tolerance limit (times)	Relative error（%）	Coexisting substances	Tolerance limit (times)	Relative error（%）
K^+	500	5.6	Ca^{2+}	1000	-7.8
Cl^-	500	6.3	PO_4^-	10	-3.3
Fe^{3+}	100	-8.7	Al^{3+}	10	-6.6
Zn^{2+}	1000	-2.4	NH_4^+	100	-5.1
Cu^{2+}	50	-3.2	Cr^{3+}	1000	4.4
Fe^{2+}	1000	6.4	Hg^{2+}	50	7.2
Mg^{2+}	100	-4.6	NO_3^-	1000	4.3
Na^+	1000	-8.4	NO_2^-	1000	5.2
SO_4^{2-}	50	-6.1	Ba^{2+}	100	-6.9
Pb^{2+}	500	-5.8	Cd^{2+}	50	7.6

Table S3 Influence of coexisting substances on RRS system

Coexisting substances	Tolerance limit (times)	Relative error（%）	Coexisting substances	Tolerance limit (times)	Relative error（%）
K^+	500	8.8	Ca^{2+}	1000	2.3
Cl^-	500	5.4	PO_4^-	10	-8.5
Fe^{3+}	500	-7.3	Al^{3+}	10	-8.7
Zn^{2+}	1000	-3.5	NH_4^+	500	-6.1
Cu^{2+}	50	-8.4	Cr^{3+}	1000	3.3
Fe^{2+}	1000	9.2	Hg^{2+}	50	5.7
Mg^{2+}	100	1.5	NO_3^-	1000	6.3
Na^+	1000	-3.7	NO_2^-	1000	1.6
SO_4^{2-}	50	-1.2	Ba^{2+}	100	-6.6
Pb^{2+}	500	-1.6	Cd^{2+}	100	5.8

Table S4 Results of RRS method

Sample	Measurement value（nmol/L）	Average (nmol/L)	Added Co^{2+} (nmol/L)	Measurement value after adding（nmol/L）	recovery（%）	RSD（%）
river water	3.56, 3.40, 3.57, 3.81, 3.65	3.60	0.5	4.13, 3.96, 4.34, 3.84, 4.39	106	4.14
waste water	15.04, 14.87, 15.34, 14.50, 14.72	14.89	0.5	15.53, 15.56, 15.34, 15.10, 15.20	91	2.14
tap water	1.27, 1.28, 1.06, 1.10, 1.17	1.18	0.5	1.36, 1.84, 1.52, 1.84, 1.70	94	8.39

Table S5 Results of SERS method

Sample	Measurement value（nmol/L）	Average (nmol/L)	Added Co^{2+} (nmol/L)	Measurement value after adding（nmol/L）	recovery（%）	RSD（%）
river water	3.92, 3.55, 3.55, 3.74, 3.63	3.68	0.5	4.24, 4.16, 4.16, 4.44, 3.92	101	4.24
waste water	15.50, 15.36, 14.25, 14.25, 14.62	14.79	0.5	15.10, 15.60, 15.36, 15.24, 14.96	92	4.06
tap water	1.25, 1.39, 1.10, 1.14, 1.24	1.22	0.5	1.72, 1.72, 1.58, 1.86, 1.70	99	9.22
